# Lombosciatique révélant un kyste péri-neural sacré

**DOI:** 10.11604/pamj.2015.21.307.7071

**Published:** 2015-08-27

**Authors:** Mouna Sghir, Nadia Lazreg

**Affiliations:** 1 Unité de Médecine Physique, CHU Tahar Sfar, Mahdia, Tunisie; 2 Service de Médecine Physique, Rééducation et Réadaptation Fonctionnelle, CHU Sahloul, Sousse, Tunisie

**Keywords:** Lombosciatique, kyste péri-neural, colonne sacrée, sciatica, peri-neural cyst, sacral spine

## Image en medicine

Les kystes péri-neuraux sont des kystes des racines nerveuses trouvés le plus souvent au niveau de la colonne sacrée survenant entre des couches de recouvrement de la périnèvre et l'endonèvre. Ils sont relativement rares et la plupart d'entre eux sont asymptomatiques. Certains kystes d'eux peuvent exercer une pression sur les éléments nerveux, entraînant d'une sciatique, d'un déficit moteur et de troubles vésicosphinctériens.Nous rapportons le cas d'une patiente âgée de 60 ans, diabétique, qui consulte pour une lombosciatique S1 bilatérale d'horaire mixte, évoluant depuis plusieurs années. L'examen trouve un syndrome rachidien et l'absence de signes de conflit disco-radiculaires. Une IRM lombaire faite a montré une formation kystique au niveau de la partie distale des racines de la queue de cheval en rapport avec un kyste péri neural. La patiente a bénéficié d'une ponction et d'une infiltration du kyste sous scanner. L’évolution est marquée par l'amélioration des lomboradiculalgies.

**Figure 1:**
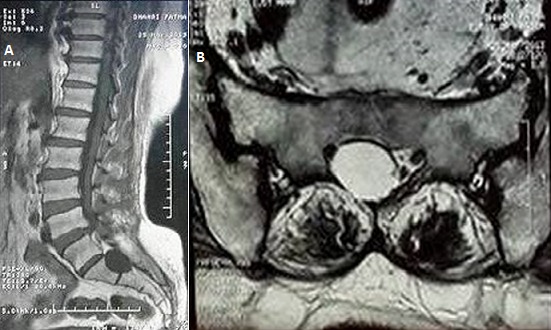
(A) IRM lombaire, coupe sagittale en T1: formation kystique au niveau de la partie distale des racines de la queue de cheval en rapport avec un kyste péri-neural; (B) IRM lombaire, coupe sagittale en T2: formation kystique au niveau de la partie distale des racines de la queue de cheval en rapport avec un kyste péri-neural

